# Hemoglobin metabolism by-products are associated with an inflammatory response in patients with hemorrhagic stroke

**DOI:** 10.5935/0103-507X.20180003

**Published:** 2018

**Authors:** Cássia Righy, Ricardo Turon, Gabriel de Freitas, André Miguel Japiassú, Hugo Caire de Castro Faria Neto, Marcelo Bozza, Marcus F. Oliveira, Fernando A. Bozza

**Affiliations:** 1 Laboratório de Pesquisa Clínica em Medicina Intensiva, Instituto Nacional de Infectologia Evandro Chagas, Fundação Oswaldo Cruz - Rio de Janeiro (RJ), Brazil.; 2 Instituto Estadual do Cérebro Paulo Niemeyer - Rio de Janeiro (RJ), Brazil.; 3 Complexo Hospitalar de Niterói - Niterói (RJ), Brazil.; 4 Hospital Universitário Antônio Pedro, Universidade Federal Fluminense - Nieterói (RJ), Brazil.; 5 Hospital Quinta D’Or - Rio de Janeiro (RJ), Brazil.; 6 Laboratório de Imunofarmacologia, Instituto Oswaldo Cruz, Fundação Oswaldo Cruz - Rio de Janeiro (RJ), Brazil.; 7 Laboratório de Inflamação e Imunidade, Departamento de Imunologia, Instituto de Microbiologia, Universidade Federal do Rio de Janeiro - Rio de Janeiro (RJ), Brazil.; 8 Laboratório de Bioquímica de Resposta ao Estresse, Instituto de Bioquímica Médica, Universidade Federal do Rio de Janeiro - Rio de Janeiro (RJ), Brazil.; 9 Instituto D’Or de Pesquisa e Ensino - Rio de Janeiro (RJ), Brazil.

**Keywords:** Iron, Heme, Cytokines, Inflammatory response, Hemopexin, Haptoglobin, Central nervous system, Subarachnoid hemorrhage

## Abstract

**Objective:**

To evaluate the relationships of brain iron and heme with the inflammatory response of the systemic and central nervous systems and to investigate the role of defensive systems against the toxicity of iron and heme in the central nervous system.

**Methods:**

We assessed a prospective cohort of patients presenting with intracerebral and subarachnoid hemorrhage. We assayed plasma and cerebrospinal fluid samples for the presence of iron, heme, hemopexin, haptoglobin, enolase, S100-β and cytokines for the first three days following hemorrhagic stroke. We also analyzed the dynamic changes in these components within both fluids and their relationship with early mortality rates.

**Results:**

Hemopexin and haptoglobin concentrations were nearly negligible in the brain after intracerebral and subarachnoid hemorrhage. Cerebrospinal fluid iron and heme concentrations correlated with a pro-inflammatory response in the central nervous system, and plasmatic and cerebrospinal fluid inflammatory profiles on the third day after hemorrhagic stroke were related to early mortality rates. Interleukin 4 levels within the cerebrospinal fluid during the first 24 hours after hemorrhagic stroke were found to be higher in survivors than in non-survivors.

**Conclusion:**

Iron and heme are associated with a pro-inflammatory response in the central nervous system following hemorrhagic stroke, and protections against hemoglobin and heme are lacking within the human brain. Patient inflammatory profiles were associated with a poorer prognosis, and local anti-inflammatory responses appeared to have a protective role.

## INTRODUCTION

Inflammatory response is a well-documented consequence of brain hemorrhage,^(1)^ and the involvement of products derived from hemoglobin metabolism contribute decisively to brain injury. In experimental studies, it is well known that iron promotes redox imbalance by mediating free radical generation^(2)^ and reducing antioxidant defenses.^(3)^ Iron has also been found to prevent DNA repair,^(4)^ augment glutamate release,^(5)^ and amplify inflammatory responses within the brain.^(6)^ In human studies, ferritin levels were correlated with poorer prognoses in intracerebral hemorrhage (ICH) patients.^(7)^

Once released from hemoglobin polypeptide chains, heme binds to many extracellular components, from phospholipid membranes^(8)^ to proteins such as albumin and hemopexin (Hx).^(9)^ Free heme has been related to an increased mortality in sepsis^(10)^ and to the severe systemic manifestations of malaria.^(11)^ Free heme can also stimulate a pro-oxidant reaction^(12,13)^ and can augment inflammatory reactions by directly stimulating toll-like receptor 4 (TLR4).^(14)^ Neurons were found to be more sensitive to the toxic effects of heme than astrocytes, which contributes to perpetuating brain injury.^(15)^

Haptoglobin (Hp) and Hx are plasma proteins synthesized by the liver that function by binding free hemoglobin and heme that have been released during intravascular hemolysis, thereby removing them from circulation. Some evidence suggests that Hp and Hx are involved in protecting the brain against injury following ICH. Following ICH, hypohaptoglobinemic and Hx-knockout mice present with higher neurological deficits and reduced striatal cell viability, respectively.^(16,17)^

Despite ample experimental inquiry, the roles of iron and heme in the pathophysiology of human brain injury after hemorrhagic stroke, as well as the potential protective mechanisms provided by Hp and Hx, are not fully understood. In this study, we aimed to evaluate the role of blood-derived products in the pathophysiology of brain injury following brain hemorrhage. We also strove to clarify the *central nervous system* (CNS)-based mechanisms of protection against iron- and heme-induced damage and the relationships among inflammatory and blood metabolism parameters with regard to early mortality rates.

## METHODS

Approval for the study was obtained from the local Ethics Committees of *Hospital Copa D'Or, Hospital Quinta D'Or* and *Complexo Hospitalar de Niterói*. In all cases, written informed consent was obtained from the patient or a surrogate.

Fifteen patients with CT-documented ICH or subarachnoid hemorrhage (SAH) admitted to the neurocritical care units at *Hospital Copa D'Or, Hospital Quinta D'Or* or *Complexo Hospitalar de Niterói* (Rio de Janeiro, Brazil) were included. The eligibility criterion was spontaneous ICH or SAH with both intraventricular hemorrhage and external ventricular device insertion within 24 hours following the onset of symptoms. Exclusion criteria were as follows: aged less than 18 years, diagnosed with pregnancy, or expected to survive less than 48 hours after admission.

Demographic information of each patient was recorded upon admission. The severity of illness in the SAH patients was assessed by calculating the Simplified Acute Physiology Score (SAPS) II, as well as the Glasgow Coma, Hunt-Hess and Fisher scales. In the ICH patients, hematoma volume was used to assess illness severity. Clinical information (heart rate, blood pressure, intracranial pressure, and cerebral perfusion pressure) and laboratory results (blood count, electrolytes, liver, and kidney function parameters) were recorded sequentially. The primary outcome was 7-day mortality.

Blood and cerebrospinal fluid (CSF) were collected at 24, 48 and 72 hours following intensive care unit (ICU) admission. Blood was collected from either an arterial line or a peripheral vein, and CSF was collected from the external ventricular device. Blood and CSF samples were assayed for cytokines, iron, heme, Hx, Hp, S100-β and enolase concentrations. We also determined the levels of CRP-t, d-dimer, fibrinogen, prothrombin and prothrombin total time in the blood, as well as the cell count, glucose and protein concentration in the CSF.

Plasma and CSF samples were centrifuged at 800g for 15 minutes at 4ºC, and the supernatant was aliquoted and stored at -70ºC until analysis. A multiplex cytokine kit (interleukin - IL-1β, IL-2, IL-4, IL-5, IL-6, IL-7, IL-8, IL- 10, IL-12, IL-13, IL-17, interferon gamma, IP-10, granulocyte colony-stimulating factor [G-CSF], granulocyte/macrophage colony-stimulating factor, monocyte chemoattractant protein 1 [MCP-1], macrophage inflammatory protein 1 [MIP-1] and tumor necrosis factor α [TNF-α]) was used according to the manufacturer's instructions (Bio-Rad, Hercules, CA, USA). Only the cytokines that were recoverable from more than 70% of the samples were analyzed.^(18)^

Iron was measured using a colorimetric assay previously described by Carter.^(19)^ Briefly, iron is simultaneously released from protein and reduced by hydrochloric-thioglycolic acid. The ferrous dialysate reacts with buffered ferrozene, a monosodium salt of 3-(2-pyridyl)-5,6-bis-(4-phenylsulfonic acid), at a controlled pH and is then measured colorimetrically at 560nm. Total heme levels were measured using a chromogenic assay (GenWay Biotech, San Diego, CA, USA), which utilizes peroxidase activity in the presence of heme to convert a colorless probe to a strongly colored (λ = 570nm) compound. Trace amounts of heme can be quantified in the 5 - 160pg (10 - 250fmol) range.

Hemopexin, Hp, enolase and S100-β concentrations were measured using a commercial enzyme-linked immunosorbent assay (ELISA) kit according to the manufacturer's instructions (LifeSciences, Newberg, OR). In this assay, any Hx, Hp, enolase or S100-β present in the samples reacts with their respective antibodies, which have been adsorbed to the surface of the polystyrene microtiter wells. Then, these antibodies conjugated with horseradish peroxidase are added. The enzyme bound to the immunosorbent is then assayed with the addition of a chromogenic substrate, 3,3',5,5'-tetramethylbenzidine, and measured at 450 nm.

### Statistical analysis

Statistical analyses were performed using Statistical Package for Social Science (SPSS) for Windows 17.0 (SPSS Inc., Chicago, IL, USA) and GraphPad Prism version 6.0 for Mac (GraphPad Software, San Diego, CA, USA). Numeric variables are expressed as the median values (interquartile range) and were assessed using the Mann-Whitney U-test and the Kruskal-Wallis test. Dichotomous variables were analyzed using χ^2^ and Fisher's exact test (with Yates correction, as indicated). Spearman analysis was employed to detect correlations among continuous variables.

## RESULTS

Of the 15 patients included in this study, 6 (40%) died within the first 7 days after ICU admission. All patients were on mechanical ventilation upon admission to the ICU, and 11 (73.3%) were using vasoactive amines (Table 1).

**Table 1:** Patient characteristics

Characteristics	All patients (n = 15)
Age (years)	59 (55 - 65)
Male gender	6 (40)
Glasgow coma scale at admission	7 (6 - 9)
Subarachnoid hemorrhage	10 (66.6)
Hemorrhagic stroke	5 (33.3)
SAPS II	43 (32 - 53)
Mechanical ventilation at admission	15 (100)
Shock at admission	11 (73.3)
7-day mortality	6 (40)
Hospital mortality	11 (73.3)

SAPS - Simplified Acute Physiology Score II. Unless otherwise stated, values are expressed as N (%) and median (interquartile range).

### Concentrations of iron, heme, hemopexin and haptoglobin in plasma and cerebrospinal fluid

We measured the concentrations of iron, heme, Hx and Hp in the plasmatic and CSF compartments throughout the first 3 days following hemorrhagic stroke. Interestingly, Hx and Hp levels were almost undetectable within the CSF and significantly lower than in the plasma during the first three days following the event. Furthermore, their concentrations did not increase during the early phase of hemorrhagic stroke. These findings suggest that these protection mechanisms against hemoglobin and heme are deficient within the brain parenchyma (Table 2).

**Table 2:** Comparison between plasma and cerebrospinal fluid iron, heme, hemopexin and haptoglobin concentrations

	24 hours		48 hours		72 hours	
	Plasma	CSF	Plasma	CSF	Plasma	CSF
Iron (mg/dL)	243.4 (137.8 - 459.1)	50.93 (34.01 - 73.62)*	74.85 (53.04 - 244.9)	37.76 (32.92 - 170.2)	94.4 (3.67 - 167.3)	54.99 (43.57 - 72.26)*
Heme (nM)	628 (587 - 1125)	599.9 (591.9 - 643.8)	604.7 (583.4 - 633.2)	613.5 (591.7 - 745.9)	630.7 (594.8 - 658.8)	682.4 (639.6 - 1093)
Hemopexin (mg/dL)	46.11 (25.02 - 78.47)	0.95 (0 - 8.0)*	50.45 (17.16 - 113.8)	0 (0 - 2.82)*	30.27 (15.46 - 65.37)	0.07 (0 - 4.74)*
Haptoglobin (mg/dL)	72.4 (42.9 - 156.3)	0.595 (0 - 4.898)*	109.3 (43.52 - 245.5)	0.865 (0 - 5.853)*	93.72 (59.18 - 205.5)	1.275 (0 - 6.175)*

CSF - cerebrospinal fluid. Values are expressed by median (interquartile range).

* Significant value (p< 0.05). Mann-Whitney test was performed between plasma and cerebrospinal fluid groups at different time points.

We identified a decrease in the concentration of plasmatic iron at 48 hours after hemorrhagic stroke, which remained stable 72 hours after the ictus (243.4 *versus* 74.85 *versus* 94.4mg/dL; p = 0.02). Concentrations of heme, Hx and Hp during the first three days after the event remained stable (Table 3).

**Table 3:** Plasmatic and cerebrospinal fluid iron, heme, hemopexin and haptoglobin kinetics during the first three days following hemorrhagic stroke

	24 hours	48 hours	72 hours
Plasma			
Iron (mg/dL)	243.4 (137.8 - 459.1)	74.85 (53.04 - 244.9)*	94.4 (3.67 - 167.3)
Heme (nM)	628 (587 - 1125)	604.7(583.4 - 633.2)	630.7 (594.8 - 658.8)
Hemopexin (mg/dL)	46.11 (25.02 - 78.47)	50.45 (17.16 - 113.8)	30.26 (15.46 - 65.37)
Haptoglobin (mg/dL)	72.4 (42.9 - 156.3)	109.3 (43.52 - 245.5)	97.32 (59.18 - 205.5)
Cerebrospinal fluid			
Iron (mg/dL)	50.93 (34.01 - 73.62)	37.76 (32.92 - 170.2)	54.99 (43.57 - 72.26)
Heme (nM)	599.9 (591.9 - 643.8)	613.5 (591.7 - 745.9)	682.4 (639.6 - 1093)
Hemopexin (mg/dL)	0.95 (0 - 8.0)	0 (0 - 2.82)	0.07 (0 - 4.74)
Haptoglobin (mg/dL)	0.59 (0 - 4.89)	0.86 (0 - 5.85)	1.27 (0 - 6.17)

Values are expressed by median (interquartile range).

* Significant value (p < 0.05); plasmatic iron concentration at 48 hours was significantly lower than that at 24 hours. Reference ranges: plasmatic iron, 0.055 - 0.16mg/dL; cerebrospinal fluid iron, 0.01 - 0.02pg/mL; plasmatic heme, 119.34 ± 6.12mg/dL; cerebrospinal fluid heme, negligible.

Upon comparing plasmatic and CSF concentrations of iron, heme, Hx and Hp, we found that the iron concentration of plasma was significantly higher than that of CSF at 24 hours and 72 hours after hemorrhagic stroke. We found no measurable differences in the concentrations of heme.

### The relationships of iron and heme with plasmatic and cerebrospinal fluid cytokines

We analyzed the correlations among iron, heme and cytokine concentrations in plasma and CSF. There was a moderate negative correlation between levels of plasmatic iron at 24 hours after the event and the plasmatic concentration of IP-10 at 72 hours after hemorrhagic stroke (r = -0.67; p = 0.025). Interestingly, there was a strong correlation between the CSF concentrations of iron at 48 hours after the ictus and the CSF IP-10 levels at 72 hours after the event (r = 0.97; p = 0.03).

There was a strong correlation between the CSF levels of heme during the first 24 hours after the ictus and the MIP-1b concentration at 48 hours after hemorrhagic stroke (r = 0.76; p = 0.01). The CSF heme concentration in the first 48 hours after the event was also negatively correlated to the CSF MCP-1 levels at 72 hours after the ictus (r = -0.82; p = 0.03). Combined, these data suggest that iron and heme are associated with an inflammatory response within the human brain after a hemorrhagic event. We found no correlation between iron or heme concentrations and other cytokines throughout the study period.

### The concentration of brain injury biomarkers in plasma and cerebrospinal fluid

In evaluating the kinetics of brain injury biomarkers, we measured a steady increase in plasmatic enolase concentrations during the first three days following hemorrhagic stroke (2.65 *versus* 4.85 *versus* 38.06mg/dL; p = 0.02). In parallel, CSF enolase concentrations progressively decreased in the first 72 hours after the ictus (16.42 *versus* 4.24 *versus* 2.82; p = 0.03). These results suggest a preferential death of neurons *versus* astrocytes, with subsequent antigen spillover from the CSF into the blood. Surprisingly, we found no change in the S100-β concentration kinetics in either the plasmatic compartment or the CSF.

### The determinants of early mortality after hemorrhagic stroke

In assessing patients over the first 7 days after hemorrhagic stroke, we found that plasmatic iron and heme concentrations were higher during the first 48 hours following the ictus in non-survivors than in survivors (iron: 496.04 *versus* 58.5mg/dL, p = 0.05; heme: 624.3 *versus* 584.7nM, p = 0.04). This finding suggests that iron overload may contribute to brain injury and early mortality. There was no observed difference between survivors and non-survivors regarding Hx or Hp concentrations throughout the first 3 days after hemorrhagic stroke, in either plasma or CSF.

Systemic (IL-6 and IL-8) and CNS inflammatory profiles (cytometry, lymphocyte and polymorphonuclear count) measured at 72 hours after the event exhibited a consistent relationship with 7-day mortality rates (Table 4).

**Table 4:** The relationship between inflammatory profiles and 7-day mortality rates three days after hemorrhagic stroke

	Survivors (n = 9)	Non-survivors (n = 6)
Plasma		
IL-6 (pg/dL)	26.15 (0.001 - 109.8)	1,271 (250.7 - 4,180)*
IL-8 (pg/dL)	3.83 (0.001 - 17.64)	134.8 (19.16 - 4,062)*
Cerebrospinal fluid		
Cytometry (cells/mm^3^)	6 (5 - 9.25)	237 (140 - 1,078)*
Red blood cell (cells/mm^3^)	13,250 (3,815 - 18,406,25)	17,685 (9,619.5 - 34,652.5)
PMN (cells/mm^3^)	0 (0 - 0)	58 (18.75 - 680.25)*
Lymphocytes (cells/mm^3^)	5 (3.5 - 6.5)	179 (121.25 - 397.75)*
Glucose (mg/dL)	69 (57.75 - 77.25)	100 (36.5 - 106)
Protein (mg/dL)	58.55 (44.47 - 92.2)	54 (38.75 - 64.75)

IL - interleukin; PMN - polymorphonuclear leukocyte. Values are expressed by median (interquartile range).

* Significant value (p < 0.05). Mann-Whitney test was performed to compare survivors and non-survivors.

We analyzed the concentrations of IL-1b, IL-2, IL-6, IL-8, GM-CSF, IP-10, MIP-1a, MIP-1b, IP-10 and RANTES in plasma. For CSF, in addition to these cytokines, we evaluated IL-4 and FGF levels. Three days after the ictus, plasmatic IL-6 and IL-8 were found to be significantly higher in non-survivors than in survivors (IL-6: 1271 *versus* 26.15pg/mL, p = 0.04; IL-8: 134.8 *versus* 3.83pg/mL; p = 0.). Conversely, local anti-inflammatory responses seem to exert a protective role. In CSF, IL-4 was found to be higher in survivors than in non-survivors during the first 24 hours following hemorrhagic stroke (34.98 *versus* 0.001pg/mL; p = 0.04). There were no measurable differences between survivors and non-survivors among the remaining cytokines in either plasma or CSF. Surprisingly, there were also no differences between survivors and non-survivors with respect to enolase and S100-β concentrations in either plasma or CSF.

## DISCUSSION

The aim of this study was to evaluate the role of blood-derived products and the protective mechanisms against hemoglobin and heme in the pathophysiology of brain injury following hemorrhagic stroke. We also attempted to determine whether iron, heme, Hx and Hp are related to patient inflammatory responses and 7-day mortality rates. Our primary findings were as follows: a deficient defense against hemoglobin-derived injury is due to virtually negligible concentrations of Hx and Hp in CSF compared to those in plasma; iron and heme were associated with an inflammatory response that triggers CSF IP-10 and MIP-1b release; and systemic iron overload was correlated with poorer prognosis; and systemic and CSF inflammatory profiles at 72 hours after the event were related to early mortality, local anti-inflammatory responses may exert a protective role following hemorrhagic stroke and SAH.

In this study, we discovered that Hx and Hp concentrations are nearly negligible within CSF compared to those in plasma and do not increase during the first 3 days after the event. Although Hp levels have already been correlated with lower mortality rates in septic patients,^(20)^ this protection seems to be lacking in the human brain. These data cast doubts on the degree of Hb and heme scavenging that occur in the human brain and agree with Galea et al., who found that most Hb were not bound to Hp. This finding suggested that the CD163-Hb-Hp system is saturated and that the primary route for Hb clearance from the CNS is by freely crossing the blood brain barrier across a concentration gradient.^(21)^ Therefore, we can conclude that the human brain lacks mechanisms against hemoglobin and heme toxicity, making the CNS more vulnerable to the toxic effects of hemoglobin degradation products.

In our study, IP-10 and MCP-1 concentrations were strongly correlated with iron and heme levels, respectively. The temporal association of iron and heme levels and the release of IP-10 and MCP-1 might suggest a causal relationship. However, IP-10 and MCP-1 release may also be induced by another element (such as thrombin). Iron and heme serve only as markers of the extension of bleeding.

The burden of systemic inflammatory response as a factor governing poor prognosis is well known in hemorrhagic stroke patients. Systemic inflammatory response syndrome is observed in up to one-third of patients with SAH and is related to extracerebral organ dysfunction and poorer patient outcomes.^(22)^ Inflammatory response components, such as fever and leukocytosis, are markers of increased mortality,^(23,24)^ and the frequency of inflammatory response parallels the severity of cerebral insult; it is more common and with a greater degree of higher-grade radiographic and clinical SAH. Both the surge in ICP^(25)^ and sympathetic nervous system activation^(26)^ may be contributing factors to this strong relationship between the severity of SAH and the degree of systemic inflammatory response syndrome. In our study, we found that increased concentrations of IL-6 and IL-8 in plasma were related to mortality. However, a local anti-inflammatory response, as shown by increased CSF IL-4 levels, served as a protective factor. The finding that CSF IL-4 is related to survival after hemorrhagic stroke agrees with other studies that show a neuroprotective role of anti-inflammatory activity following brain injury.^(27,28)^

The limitations of our study include the small sample size and the mixed profile of patients with ICH and SAH. Despite this heterogeneity in the root causes of ICH, iron and heme still play a key role in the pathophysiology of both diseases.

## CONCLUSION

This study provides preliminary evidence that iron and heme play a role in triggering inflammatory response in the CNS and that the human brain lacks protection mechanisms against hemoglobin by-products. Moreover, our study reinforces the concept that systemic inflammatory response syndrome. is an important determinant of the outcome of hemorrhagic stroke patients. More extensive clinical studies of these biomarkers will be required to define their mechanistic and prognostic roles following hemorrhagic stroke.
